# The Arg233Lys AQP0 Mutation Disturbs Aquaporin0-Calmodulin Interaction Causing Polymorphic Congenital Cataract

**DOI:** 10.1371/journal.pone.0037637

**Published:** 2012-05-25

**Authors:** Shanshan Hu, Binbin Wang, Yanhua Qi, Hui Lin

**Affiliations:** 1 Department of Ophthalmology, the 2nd Affiliated Hospital of Harbin Medical University, Harbin, China; 2 Center for Genetics, National Research Institute for Family Planning, Beijing, China; University of Arkansas for Medical Sciences, United States of America

## Abstract

Calmodulin (CaM) directly interacts with the aquaporin 0 (AQP0) C-terminus in a calcium dependent manner to regulate the water permeability of AQP0. We previously identified a missense mutation (p.R233K) in the putative CaM binding domain of AQP0 C-terminus in a congenital cataract family. This study was aimed at exploring the potential pathogenesis of this mutation causative of cataract and mainly identifying how it influenced the binding of AQP0 to CaM. Wild type and R233K mutant AQP0 with EGFP-tag were transfected separately into Hela cells to determine the expression and subcellular localizations. The co-immunoprecipitation (CoIP) assay was used to detect the interaction between AQP0 and CaM. AQP0 C-terminus peptides were synthesized with and without R233K, and the binding abilities of these peptides to CaM were assessed using a fluorescence binding assay. Localizations of wild type and R233K mutant AQP0 were determined from EGFP fluorescence, and the chimeric proteins were both localized abundantly in the plasma membrane. Protein expression levels of the culture cells showed no significant difference between them. The results from CoIP assay implied that R233K mutant presented more weakly in association with CaM than wild type AQP0. The AQP0 C-terminal mutant peptide was found to have 2.5-fold lower binding affinity to CaM than wild type peptide. These results suggested that R233K mutation did not affect the expression, location and trafficking of the protein but did influence the interaction between AQP0 and CaM. The binding affinity of AQP0 C-terminus to CaM was significantly reduced. Due to lack of the modulation of the Ca2+-calmodulin complex, the water permeability of AQP0 was subsequently augmented, which might lead to the development of this cataract.

## Introduction

Aquaporins (AQPs) are integral membrane proteins that are ubiquitous transmembrane channels to facilitate the diffusion of water and/or selected small neutral solutes, such as urea and glycerin, across the membrane of the cell [1]. In mammalians, 13 isoforms (AQP0-12) have been identified in various tissues [2]. Among these isoforms, two aquaporins are expressed in lens and play a critical role for maintaining lens transparency. AQP1 is expressed in lens epithelial cells, while AQP0 is abundant in lens fibers, which contributes to over 50% of total membrane proteins of the fibers [3]. These two aquaporins are virtually impermeable to anything but water. [2].

Although relatively low in water permeability per molecule, compared with other aquaporins, AQP0 is present at high concentrations in the lens [4]. The high concentrations of AQP0 are gradually accumulated during the procedure that new lens fibers are continuously differentiated from epithelial cells and subsequently turn into mature lens fibers [5]. So it provides the major permeability pathway of water in lens fibers [4]. As an avascular tissue, the lens (particular the center of the lens) relies heavily on this transport system to preserve the normal flow of water across the cells to maintain lens transparency and homeostasis [5].

AQP0, also designated as major intrinsic protein (MIP), is a 28.2 KDa protein with 263 amino acids. It possesses six transmembrane domains and intracellular NH_2_- and COOH- terminals (residue 220–263) and assembles as a tetramer containing four water pores. Each monomer functions independently as a water channel [6]. It has been revealed that Ca2+ is an important factor in the modulation of the water flow of AQP0, but not the water permeability of AQP1 [4].The water permeability is modulated by the alteration of calcium concentration through the Ca2+-calmodulin complex binding to the C-terminus specific domain (residue 223–242) of AQP0 [7]. Increasing Ca^2+^ promotes the binding of CaM, which accordingly lowers the water permeable ability of channels [4].

Calmodulin (CaM) is the archetype of the calcium-modulated protein family. It intracellularly binds calcium with high affinity and specificity and modulates the activity of a wide range of channels and enzymes in response to calcium signals, including aquaporins [7], connexins [8], and voltage gated ion channels [9]. CaM is a small protein with only 148 amino acids, containing four EF-hand motifs that form two structurally similar domains connected by a flexible central linker. Each motif binds a calcium ion. The saturated binding of Ca2+ reorientates the two helices in each motif into a nearly perpendicular orientation and exposes the hydrophobic methyl group on the protein. The conformational changes lead to a Met-rich cavity-containing hydrophobic protein surface, which binds to a basic amphiphilic alpha-helical segment (Pep C) on the target proteins [10], [11], [12].

In our previous study, we identified that p.R233K mutation at the C-terminus of MIP/AQP0 was associated with a congenital polymorphic cataract in a six-generation family (Figure S1, S2) [13]. The mutation was just located in the CaM binding domain of the C-terminal. Hence the study presented herein was aimed to demonstrate if the shift from arginine to lysine influences the binding interaction between AQP0 and CaM and to disclose the molecular biological mechanism of this mutation causative of cataract.

## Materials and Methods

### Plasmids

The pCMV6-ENTRY expression vector containing the open reading frame of the human AQP0/MIP gene (wild type AQP0) and the pCMV6-XL5 expression vector containing the full-length sequence of the human calmodulin (CaM) gene were obtained from OriGene (Rockville, US). Difference between two expression vectors presented that the former was tagged with C-terminal Myc-tag for easy detection and purification with antibodies.

The pEGFP-n1 vector, the TK promoter pGL3-basic vector with 4×GAL4 DNA-binding sites, the Renilla luciferase control plasmid pREP7-RLuc, the pCMX-Gal4 vector containing Gal4DBD, and the pCMX-VP16 vector with potent transactivating domain of HSV VP16 were provided by Dr. Dongmei Su (National Research Institute for Family Planning, Beijing, China).

### Antibodies

Mouse anti-Myc tag antibody, rabbit anti-CaM antibody, and anti-human IgG antibody were purchased from Abcam (Cambridge, MA). Goat anti-rabbit poly-HRP and anti-mouse poly-HRP were purchased from Pierce (Rockford, US).

### Chemicals and Reagents

The Site-Directed Mutagenesis Kit was purchased from Stratagene (La Jolla, CA). The Polymerase Chain Reaction (PCR) Purification Kit and the Plasmid Midi Kit were purchased from Qiagen (Valencia, CA). Other chemicals and reagents were obtained from Sigma (St. Louis, MO).

### Site-directed mutagenesis and plasmid construction

The expression vector for R233K mutant was constructed by using site-directed mutagenesis with the following oligonucleotide primers and their complements: sense primer, 5′-CCG GCT CAA GAG TAT TTC TGA GAA ACT GTC TGT CCT C-3′; antisense primer, 5′-GAG GAC AGA CAG TTT CTC AGA AAT ACT CTT GAG CCG G-3′. DNA sequencing confirmed the produced mutation.

To create EGFP-AQP0 fusion proteins, the open reading frames of wild type and R233K mutant AQP0 were amplified by PCR with a set of two appropriate primers (sense primer, 5′-AGT ACC TCG AGA TGT GGG AAC TGC GAT CAG C-3′; antisense primer, 5′-CTA CGA AGC TTC AGG GCC TGG GTG TTC AGT T-3′). Then products were cloned into Xho I and Hind III digested pEGFP-n1 vector. The resultant plasmids were termed as pEGFP-normal and pEGFP-R233K, respectively.

Based on the sequence of human AQP0/MIP cDNA, DNA fragments encoding the C-terminus of AQP0 (amino acids 220–263) were amplified by PCR using wild type and R233K mutant AQP0 expression vectors as the template (sense primer, 5′-CCG GAA TTC GAC TTT CTT CTC TTC CCC CGG CTC-3′; antisense primer, 5′-CTA GCT AGC CTA CAG GGC CTG GGT GTT CAG TTC -3′). These produces were inserted into the expression vector pCMX-Gal4, and termed as Gal4-MIP-wt and Gal4-MIP-R233K, respectively.

The open reading frame of CaM was amplified by PCR from CaM vector and inserted into pCMX-VP16 vector to create pCMX-VP16-CaM vector (sense primer, 5′-CGC GGA TCC GGC TGA CCA CTG ACT GAA GAG C- 3′; antisense primer, 5′-CTA GCT GCT CAC TTT GCT GTC ATC ATT TGT ACA AAC TCT TC-3′). Each clone was entirely sequenced to confirmed that none mutations had been imported by PCR.

### Cell culture and transfection

Human cervical cancer (Hela) cells and human embryonic kidney (HEK 293t) cells were maintaining in DMEM (Dulbecco's modification of Eagle's medium) or IMDM (Iscove's modified Dulbecco's medium) supplemented with 10%FBS (fetal bovine serum), 100 mg/ml penicillin and 100 mg/ml streptomycin in a humidified atmosphere containing 5% CO_2_ at 37°C. Transfection was carried out using Lipofectamine 2000 (Invitrogen Corporation, Carlsbad, CA, USA) or a standard calcium phosphate method.

### Expression and Localization

Hela cells were plated in 6-well plates 24 h prior to transfection at approximately 60% confluency. The pEGFP-normal and pEGFP-R233K expression constructs were transfected separately into Hela cells using Lipofectamine 2000 according to the manufacturer's protocols. The empty vector pEGFP-n1 was transfected as a control. Forty-eight hours after transfection, the cells were analysis by fluorescence microscopy.

### Western blot analysis

After transfected with wild type AQP0 or R233K mutant AQP0 plasmids separately, HEK 293t cells were harvested and lysed in lysis buffer (50 mM Tris-HCl, 1% NP40, 150 mM NaCl, 1 mM EDTA and 1 mM PMSF) for 30 min at 4°C. Total cell extracts were separated by 12% SDS/PAGE gels, and then transferred to PVDF membranes. The membranes were incubated with anti-Myc and anti-β-actin antibodies. The signals were visualized by using the chemiluminescent substrate method with the SuperSignal West Pico Kit provided by Pierce. β-actin was used as an internal control for normalizing the loading materials.

### Co-immunoprecipitation (CoIP) assay

The HEK 293t cells were transfected with wild type and R233K AQP0 plasmids seperately. The pcDNA3.1 plasmid was transfected as a control. Forty-eight hours after transfection, cells were washed with PBS twice and lysed in lysis buffer (50 mM Tris-HCl, 1% NP40, 150 nM NaCl, 1 mM PMSF and protease inhibitor cocktail) with 2 mM CaCl_2_ (for Ca^2+^ condition) for 30 min with gentle shaking. Lysates were clarified by centrifugation at 15,000 g at 4°C for 10 min, and the supernatant was precleared with Protein G-agarose beads. Rabbit anti-CaM or mouse anti-Myc tag antibody was added for immunoprecipitation. The precipitates were then subjected to SDS-PAGE and western blotting analysis. Total cell lysates were used as input control.

### Luciferase reporter assay

5×10^4^ HEK 293t cells were seeded in 24-well plates and transfected with 500 ng TK promoter reporter plasmids and 250 ng of indicated constructs or vector alone (as a control) using a standard calcium phosphate method. The Renilla luciferase control plasmid pREP7-RLuc was co-transfected at 50 ng/well as an internal control reporter. After transfected 30 hours, cells were harvested and lysed in passive lysis buffer (Promega) with 2 mM or 4 mM CaCl_2_ (for Ca^2+^ condition). The transfection efficiency was normalized to the Renilla luciferase activity using the Dual Luciferase Reporter Assay System (Promega, Madison, WI, USA) according to the manufacturer's protocols. Every experiment was performed at least three times to eliminate handling error. Student's t test was utilized to determine the statistical significance of the experimental data.

## Results

### Subcellular localization of WT- AQP0 and R233K-AQP0

**Figure 1:**
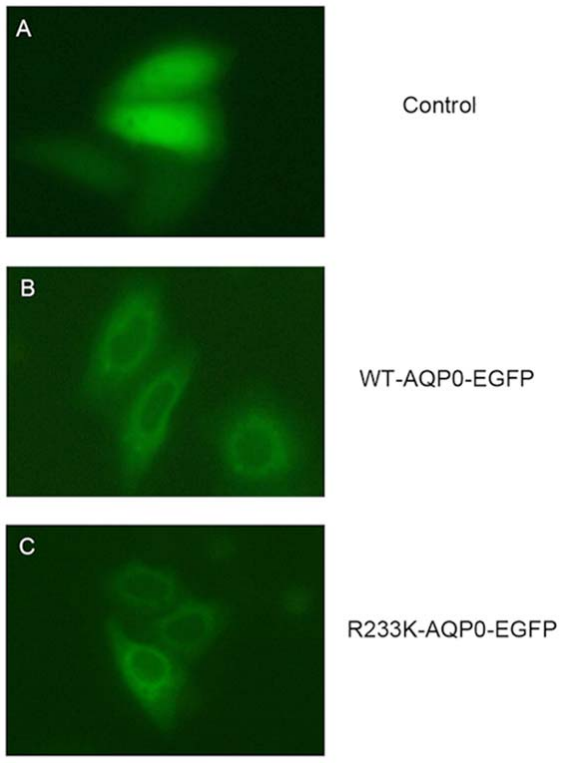
Localizations of wild type and R233K mutant AQP0 EGFP-fusion proteins. Localizations of wild type and R233K mutant AQP0 EGFP-fusion proteins in transfected Hela cells was viewed by fluorescent microscope. (A) The empty vector pEGFP-n1 was transfected as a control. Epifluorescent images of cells transfected with the WT-AQP0-EGFP (B) and R233K-AQP0-EGFP (C) showed the fusion proteins were localized abundantly in the plasma membrane of Hela cells.

Previous studies have reported that the critical amino acids at the C-terminus of AQP0 were involved in the trafficking and localization of the protein from the trans-Golgi network to the plasma membrane [14]. So we first determined whether R233K mutant would present in the plasma membrane. With the EGFP (Enhance Green Fluorescent protein) tagged, pEGFP-normal and pEGFP-R233K expression vectors were transfected separately into Hela cells and were analysed by fluorescence microscopy. As shown in Figure 1, the empty vector pEGFP-n1 (control) was located in both the nucleus and cytoplasm, while both wild type AQP0 (WT-AQP0-EGFP) and R233K mutant AQP0 (R233K-AQP0-EGFP) were localized abundantly in the plasma membrane of Hela cells. These images mainly illustrated that the substitution of lysine for arginine at position 233 in the AQP0 did not impact the trafficking of the expressed protein to the plasma membrane.

### Expression levels of R233K-AQP0 in cultured cells

**Figure 2:**
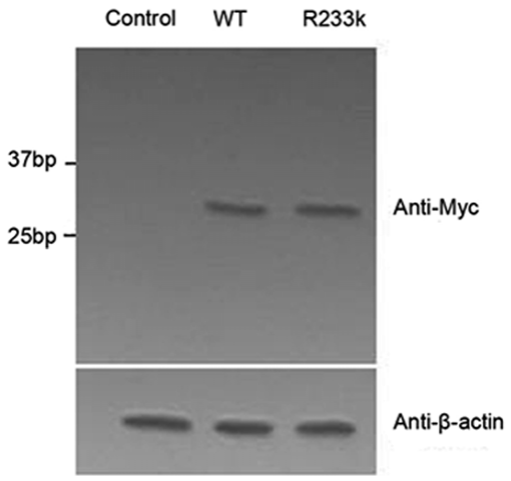
The expression levels of WT and R233K-AQP0 and the association of R233K-AQP0 and CaM. The expression levels of WT and R233K AQP0 in HEK 293t cells. Western blots were performed with the anti-Myc antibody indicated. The p.cDNA3.1 plasmid was used as a negative control. β-actin was used as the loading control.

It was reported that the level of AQP0 expressing in the plasma membrane directly affected the water permeability of the membrane. Western blot was performed by anti-Myc antibody directed against Myc-tag of AQP0-Myc proteins. A band was detected approximately at 30 KDa, which corresponded to the expression sizes of full length AQP0 combined with full length of Myc protein. The pcDNA3.1 expression plasmid was used as a negative control. Representative western blot results showed that wild type AQP0 (WT-AQP0) and R233K mutant AQP0 (R233K-AQP0) were all exogenously expressed at comparable levels in comparison with the β-actin loading control (Figure 2). That meant the expression levels of AQP0 in cultured cells were not altered by introducing this mutation.

### R233K mutation influences the interaction between AQP0 and CaM

**Figure 3:**
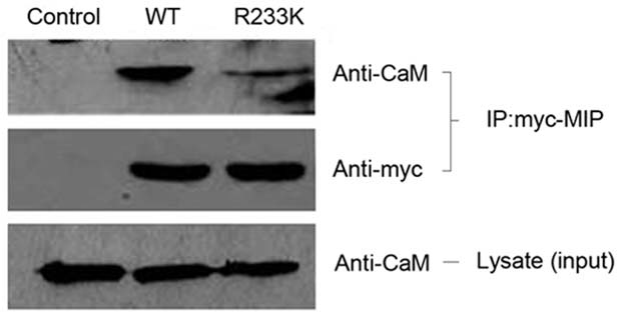
The association of R233K mutant and CaM under the Ca^2+^-dependent condition. HEK 293t cells were transfected with WT-AQP0 or R233K mutant expression plasmid tagged with –Myc tag for 48 hours. In the presence of Ca^2+^ (2 mM CaCl_2_), the whole cell extracts were prepared and immunoprecipitated with the anti-CaM antibody, and were incubated with Protein G-agarose beads. Immunoprecipitates were submitted to western blotting analysis with anti-Myc antibody. Lysate input control indicated the expression of endogenous CaM. R233K mutation of AQP0 C-terminus weakens the interaction between AQP0 and CaM.

The water permeability of AQP0 could be modulated by the Ca2+-calmodulin complex directly interacting with the C-terminus. Thus, we examined whether the formation of cataract was concerned with the alteration of interaction between CaM and AQP0 due to R233K within the CaM binding domain. To validate this assumption, we detected the association of AQP0 with CaM using a CoIP assay. Lysate input control denoted that abundance of endogenous CaM were expressed similarly in each HEK 293t cell line (Figure 3 bottom). Results with anti-Myc tag antibody revealed the same expression levels of WT-AQP0 and R233K-AQP0 (Figure 3 middle). The difference was obviously observed between the two AQP0-CaM complexes. There was merely a small amount of CaM binding to R233K-AQP0, which was dramatically decreased, compared with WT-AQP0 (Figure 3 top). Cells transfected with pcDNA3.1 expression plasmid was used as a negative control. These results signified that R233K mutation weakened the interaction between AQP0 and CaM, which infered that arginine amino acid in this region was one of governing factors in the binding process.

### The reduced binding affinity of R233K-AQP0 to CaM was measured by luciferase reporter assay

**Figure 4:**
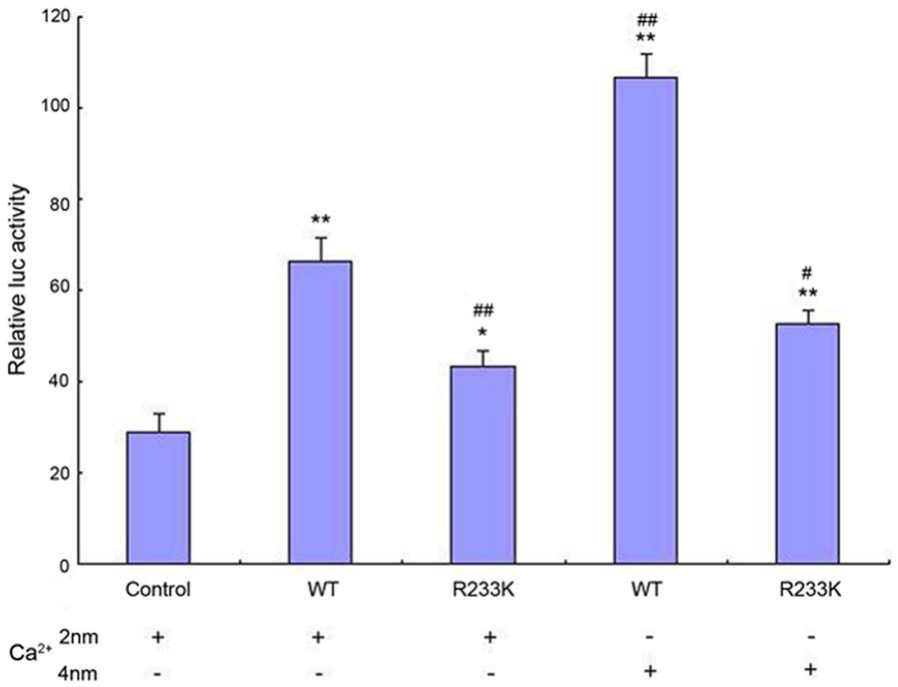
The binding affinity of R233K-AQP0 to CaM. R233K mutant decreased the binding affinity of AQP0 to CaM and was insensitive to calcium regulation. HEK 293t cells were contransfected with the TK promoter reporter plasmid, the Renilla luciferase internal control plasmid, the VP16-CaM plasmid and the indicated constructs (including Gal4-MIP-wt, Gal4-MIP-R233K, as well as empty vector pCMX-Gal4). To assess the binding affinity of R233K-AQP0 to CaM, relative luciferase activity was measured after transfected for 30 hours in the presence of different calcium concentration (2 mM or 4 mM CaCl_2_). Data were shown as mean values from four independent experiments. Significant differences were calculated using the independent-sample *t* test. *p<0.05, ** *p*<0.01 compared with empty vector pCMX-Gal4; # *p*<0.05, ## *p*<0.01 compared with Gal4-MIP-WT. Significant differences were found between wt and R233K.

To further explore to what extent this mutation decreased the binding affinity of AQP0 to CaM, luciferase reporter assay was first employed with Ca^2+^ 2 mM. According to Rose et al [15], the peptide containing residues 220–263 of AQP0 (embracing CaM binding domain 223–242) was applied in our study for experimental convenience. Representative results of luciferase reporter assay analysis suggested that a 2.5-fold upregulation of the luciferase activity presented in the cotransfected HEK 293t cells of Gal4-MIP-wt peptide and VP16-CaM, compared with Gal4-RXRa alone (Figure 4). Luciferase activity in the cotransfected cells of Gal4-MIP-R233K peptide and VP16-CaM was diminished about 36% in contrast with wild type (*t* test, p<0.005). In order to ascertain whether the binding affinity of Gal4-MIP-R233K peptide to CaM could be regulated by changing Ca2+ concentration, we increased the Ca^2+^ concentration in passive lysis buffer to 4 mM. The results displayed that the binding affinity of Gal4-MIP-wt peptide to CaM was dramatically elevated 1.5 fold. However, no more than 5% increase occurred in Gal4-MIP-R233K contransfection cells (Figure 4). These findings expressed that the mutant with R233K decreased at least one of third of the binding affinity of AQP0 to CaM and became much less sensitive to Ca2+ concentration than WT-AOP0.

## Discussion

Based on our previous findings that R233K in AQP0 was the cause of a polymorphic congenital cataract, we conducted this investigation on how the shift from arginine to lysine induced the formation of this cataract, in other words, the role of arginine amino acid in the COOH-terminus.

To date, ten known mutations in human AQP0 have been documented including seven missense mutations (R233K [13], T138R, E134G [16], R33C [17], V107I [18], Y177C [19], and R187C [20]), one deletion mutation (Δ213-AQP0) [21], one splice-site mutation (IVS3 -1 G>A) [22] and an initiation code mutation (Met1?) [23]. These mutations except for R233K were all speculated to be correlated to the trafficking or localization of the protein. Among them, only Δ213-AQP0 and R233K affected the COOH-terminus of AQP0. The former shifted the frame of the whole C-terminus, which created a premature and shortened protein that led to the failure of the trafficking of protein and the lowering of the water permeability [24]. Furthermore, according to the study of Golestaneh, et al, phosphorylation at serine231 and 235 of APQ0 was required for proper intracellular transport of MIP/AQP0 from the trans-Golgi network to the plasma membrane [14]. These studies pinpointed some critical amino acids in C-terminal domain was responsible for normal trafficking and localization of the protein in the plasma membrane. While in our study, it was clearly noticed that R233K mutant was localized as abundantly as WT-AQP0 in the plasma membrane of cultured cells, which demonstrated that the substitution of lysine for arginine at position 233 did not impact AQP0 trafficking to the plasma membrane. Obviously, R233K was the only natural human AQP0 mutation that had no relation to the protein trafficking and aggregation in the cytoplasm, which lured us to pursue further study.

AQP0 was the most abundant membrane protein in lens fibers. Lens development was highly sensitive to dosage/timly expression level of AQP0. Some findings suggested that the reduced AQP0 expression could be associated with lens abnormalities [25]; therefore, western blots were used to examine the expression level of R233K mutant in this study. The results elucidated that WT-AQP0 and R233K-AQP0 were all exogenously expressed at comparable levels, compared with the β-actin control. It hinted that Arg233 was not involved in the protein expression. It was important to note that there were no differences in dosage expression between WT-AQP0 and R233K-AQP0 before performing further experiments.

AQP0 begins to express in the primary fiber cells at the earlier stage of embryonic development, which indicates that it is essential to normal lens development in vertebrates. Although several presumptive roles of AQP0 in vivo have been reported, only the water permeability and cell-to-cell adhesion were authentically proven 26,27. AQP0 is a less efficient water channel than AQP1 [28], but as the only water channel protein expressing in the plasma membranes of lens fiber cells, it is responsible for approximate 80% of the water permeability of lens fiber cells [29]. So water transport is one of the major roles of AQP0.

A great quantity of evidence from functional studies identify that the water permeability of AQP0 is regulated by PH and calcium concentration [5], [30], [31]. Calcium acts through calmodulin to perform regulation [5]. CaM may be inhibitory to channel permeability by capping the vestibules of two monomers within the AQP0 tetramer, which reduces the water transport ability of APQ0 [32]. As the aforementioned, calmodulin with the saturated binding of Ca2+ (each motif binds a calcium ion) has a Met-rich cavity-containing hydrophobic surface and has to bind to a basic amphiphilic alpha-helical segment (Pep C) to function. The residues 223-242 of AQP0 C-terminus is a putative CaM binding site, containing a short α-helix (residues 230–238) which consists of basic amphiphilic amino acids (Ile-Ser-Glu-Arg-Leu-Ser-Val-Leu-Lys) [33], [34]. Analysis of the properties of these amino acids reveals that this peptide embraces both hydrophobic (Ile/Leu/Val) and hydrophilic (Ser/Glu/Arg/Lys) amino acids and the basic property in this area is mainly produced by Arg233. Arginine has a complex guanidinium group (Figure S3A) at one of its distal ends. It makes ariginine always positively charged in neutral, acidic and basic conditions. The positive charge can be delocalized and form multiple hydrogen bonds. That is why arignine has very strong binding ability and always appears in the critical connection region of protein. Actually, lysine has very similar properties with arginine. They can substitute for each other without causing any disfunction in some areas [35]. But as shown in this study, the replacement of Arg233 by Lys led to severely functional impairment of APQ0. It was most likely because during the binding process, arginine could provide an extra hydrogen bond via one of guanidiano nitragens than lysine that only had a single ε-amino group (Figure S3B), which was proved by a three-dimensional structure study [36].

Thus, the replacement of Arg233 by Lys made the binding to CaM less stable, impaired the binding affinity of APQ0. Moreover, when we elevated calcium concentration up to 4 mM, almost 2-fold increasing of luciferase activity was present in WT-AQP0 cells than R233K-AQP0 cells. Without sufficient binding to CaM, R233K mutant was deprived of the efficient modulation of the internal Ca2+ signal. Consequently, the intracellular water homeostasis was disrupted, which caused lens fiber cells to turn into a high water permeable state. Hereby hydropic degeneration was most likely reason why cataract was developed in this family. It was apparently concluded that the vital role of Arg233 in the C-terminal was to provide the basic property to the domain and enough hydrogen bonds to bind to CaM.

## Supporting Information

Figure S1 **A six generation pedigree with autosomal dominant congenital cataract.**
(TIF)

Figure S2 **Binocular slit lamp photographs of polymorphic cataract phenotype presented in this family.** A: Fine punctate opacities in the posterior cortex; B: Anterior polar cataract with punctate opacities in the anterior cortex; C: Punctate opacities in the cortex and core of the lens; D: A mass of irregular opacification clustering in the anterior cortex.
(TIF)

Figure S3 **Chemical structure of arginine (A) and lysine (B).**
(TIF)
