# Supplementary figures and images for: The Arg233Lys AQP0 Mutation Disturbs Aquaporin0-Calmodulin Interaction Causing Polymorphic Congenital Cataract
Source: PLoS One. 2012 May 25;7(5):e37637. doi: 10.1371/journal.pone.0037637 (PMC3360748; doi:10.1371/journal.pone.0037637)

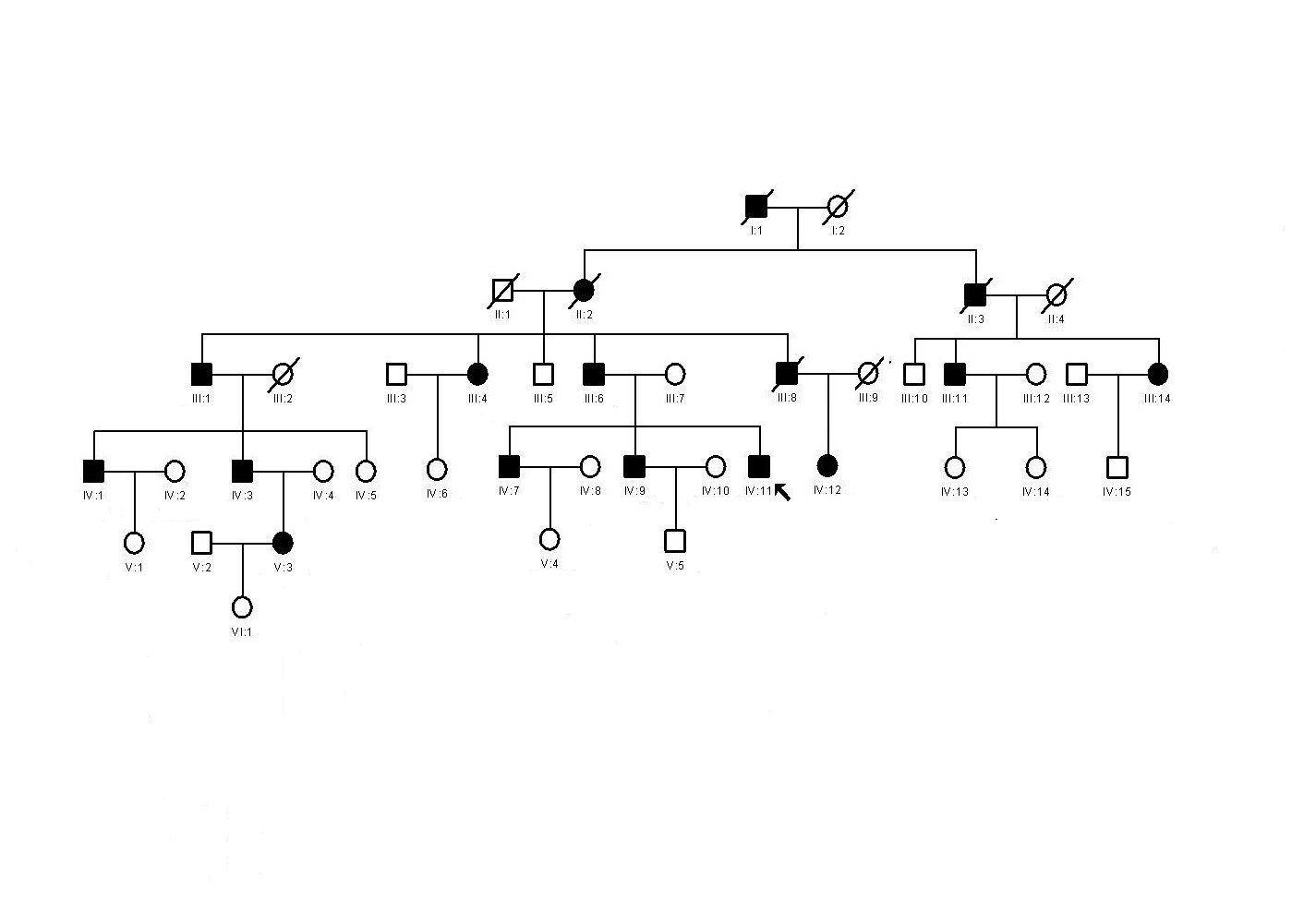

Supplement: Figure S1 — A six generation pedigree with autosomal dominant congenital cataract. (TIF) [file pone.0037637.s001.tif]

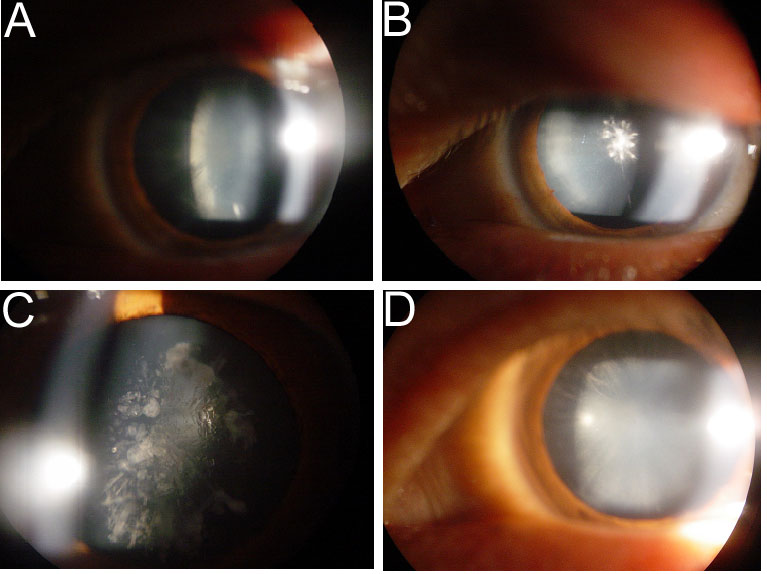

Supplement: Figure S2 — Binocular slit lamp photographs of polymorphic cataract phenotype presented in this family. A: Fine punctate opacities in the posterior cortex; B: Anterior polar cataract with punctate opacities in the anterior cortex; C: Punctate opacities in the cortex and core of the lens; D: A mass of irregular opacification clustering in the anterior cortex. (TIF) [file pone.0037637.s002.tif]

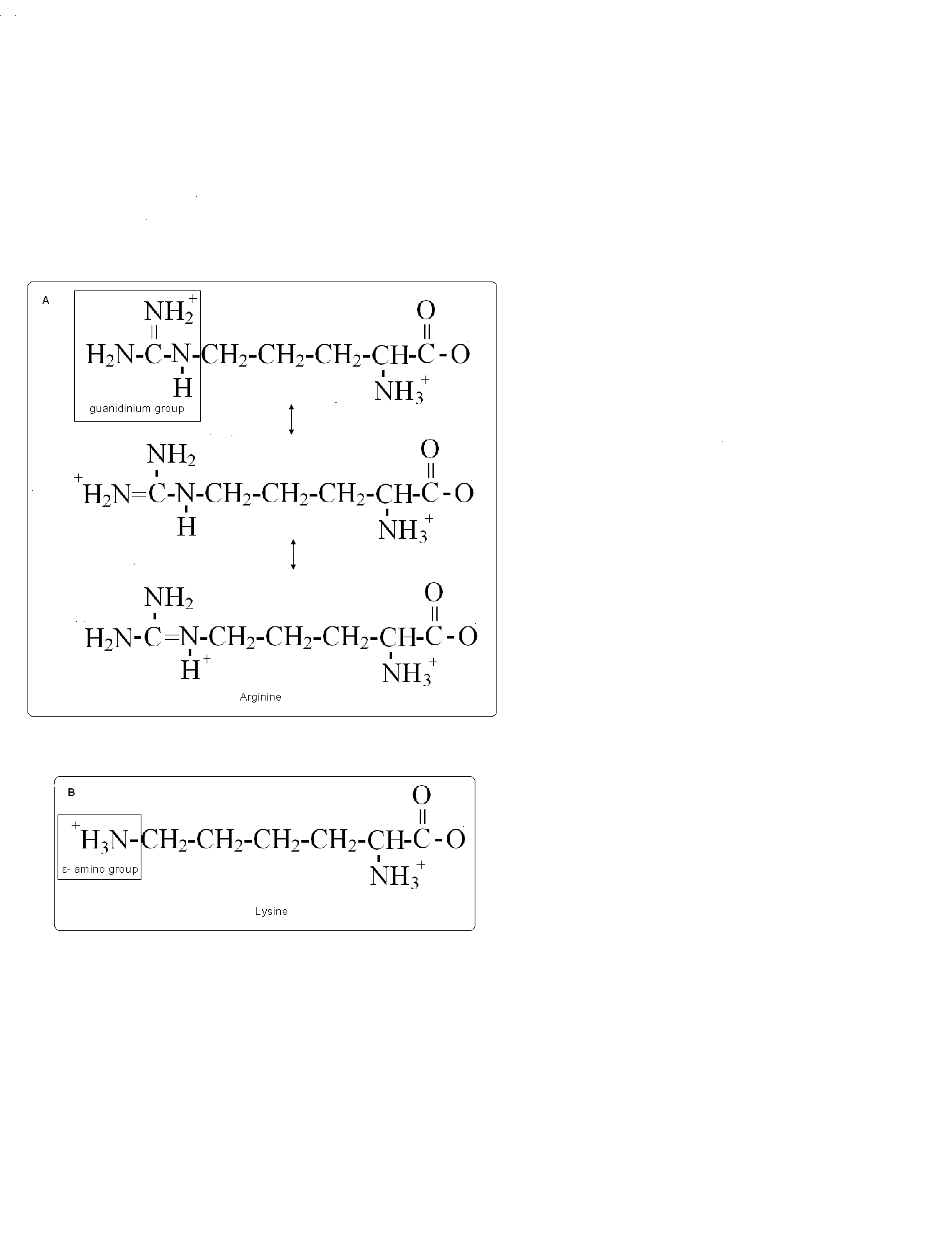

Supplement: Figure S3 — Chemical structure of arginine (A) and lysine (B). (TIF) [file pone.0037637.s003.tif]
